# Research on Cracking Causes of Industrial Roof Slabs and Optimized CFRP Strengthening Measures

**DOI:** 10.3390/ma19214625

**Published:** 2026-10-30

**Authors:** Bo Yang, Fan Bu, Xudong Chen

**Affiliations:** 1 School of Architectural Engineering, Anhui University of Applied Technology, Hefei 230011, China; 2 Anhui No. 2 Construction Engineering Quality Supervision and Inspection Station Co., Ltd., Hefei 230031, China

**Keywords:** second-floor roof slabs, restrained drying shrinkage, interfacial transition zone, CFRP strengthening, grid-shaped layout, low-pressure grouting

## Abstract

Large-scale irregular cracks developed on the second-floor roof slabs of an industrial plant shortly after formwork removal. To identify the cracking mechanism and optimize the strengthening scheme, field inspection, X-ray diffraction (XRD) phase analysis, restrained-shrinkage stress calculation, and nonlinear finite element (FE) simulation were combined in a closed-loop study. The concrete strength, reinforcement arrangement, slab thickness, and flexural capacity all met the specification requirements, and XRD detected no crystalline sulfate-bearing phases within the 1–2 wt.% detection limit, ruling out structural overloading and sulfate-type chemical attack. The cracking was attributed mainly to excessive restrained drying-shrinkage stress caused by mix-proportion defects (a high water-to-binder ratio, excessive fly ash, and a high mud content in the fine aggregate); the calculated maximum restrained shrinkage stress of 3.19 MPa exceeded the concrete tensile strength. Preferential microcrack initiation in the aggregate–mortar interfacial transition zone (ITZ) is presented as a proposed mesoscale mechanism, because direct microstructural (SEM) observation was not performed. Under identical total CFRP consumption, a four-dimensional evaluation (bearing capacity, material utilization, crack suppression, and relative interfacial slip) showed that the grid-shaped layout outperformed the cross- and hash-shaped layouts, and a ±10% sensitivity analysis kept all predicted crack-width variations within ±5%. A composite remediation of low-pressure grouting plus grid-shaped CFRP was then applied; after 12 months of monitoring, the maximum crack width decreased from 0.32 to 0.04 mm, demonstrating the synergistic effect of the composite system rather than CFRP alone. This case provides a reference for the diagnosis and treatment of shrinkage cracking in comparable industrial roof slabs.

## 1. Introduction

Cracking of reinforced concrete (RC) slabs remains a frequent quality defect in the high-quality development of the construction industry [1]. It results from the coupled effects of material proportioning, construction and curing, boundary restraint, and temperature and humidity variations. Misjudging the cause of cracking readily leads to engineering disputes, so accurate identification of the crack-formation mechanism is the prerequisite for selecting a scientific treatment. Reliable crack detection and cause diagnosis are therefore the first steps in slab-defect remediation.

Both crack detection and crack-mechanism research have advanced considerably. For detection, automated vision-based systems [2], deep-learning models trained on super-resolution images [3], and unmanned aerial vehicles combined with image segmentation [4] have been developed. For shrinkage cracking of slab-type members, prevention and control strategies covering materials, detailing, and curing have been proposed [5,6,7]. At the mesoscale, in situ tests have confirmed that cracks deflect at the aggregate–mortar interface, where the coarse-aggregate distribution and the interfacial transition zone (ITZ) govern crack propagation [8]. Long-term restrained shrinkage tests [6] and the solidification–microprestress–microplane (SMM) model [9] have also been used to evaluate and predict shrinkage cracking. These studies provide effective tools for crack-cause diagnosis. Diagnosis, however, is only the first step, because the effectiveness of subsequent strengthening depends strongly on the bond between the strengthening material and the concrete.

Among the available strengthening materials, carbon fiber-reinforced polymer (CFRP) is widely used because of its high strength-to-weight ratio and excellent durability, although its effectiveness is governed critically by the interfacial bond with concrete. Surface grooving improves the bond performance of CFRP–concrete interfaces [10], and the bond mechanism of near-surface-mounted CFRP has been clarified [11]. FRP U-jackets mitigate intermediate-crack debonding in FRP-plated RC beams [12]. The degradation of CFRP–concrete bond and tensile properties under salt freeze–thaw cycles has been analyzed [13]; the temperature dependence of FRP-sheet bond and debonding has been revealed [14]; and the long-term durability of externally bonded FRP in infrastructure rehabilitation has been evaluated [15]. These studies confirm that interfacial bond performance is a key factor controlling the effectiveness of CFRP strengthening.

Early studies of CFRP-strengthened RC slabs focused on the static flexural performance of members strengthened with FRP anchors [16,17], CFRP strips [18], and externally bonded FRP sheets [19], establishing the basic flexural behavior and demonstrating the effectiveness of external bonding. Advanced materials have since been developed: externally bonded CFRP sheets [20] and embedded SMA-CFRP composite plates [21] have been used for RC members, whereas hybrid grid-reinforced UHPC [22] and textile-reinforced mortar [23] have been applied to two-way slabs. For specific loading conditions, FE models of FRP-strengthened RC beams under impact loading have been established [24], and the nonlinear behavior of RC slabs strengthened with prestressed CFRP strips under high-cycle fatigue has been analyzed [25]. Textile-reinforced mortar has also been reviewed for strengthening deficient RC columns [26].

Most existing studies address either the concrete cracking mechanism or CFRP strengthening in isolation, and strengthening effectiveness is commonly evaluated with a single indicator such as load capacity or deflection. For cracked two-way floor slabs in engineering practice, however, complete closed-loop case studies that integrate field inspection, quantitative crack-cause analysis, simulation-based scheme optimization, on-site remediation, and long-term monitoring remain scarce.

To fill this gap, this paper presents a slab-crack diagnosis and strengthening study of a factory building. Beyond documenting a successful repair, the scientific novelty lies in three aspects:

(1) New mechanistic insight: rather than attributing the cracking to generic shrinkage, the macroscopic restrained shrinkage stress is linked quantitatively to the mesoscopic ITZ tensile strength, providing a mechanical threshold for the initiation of non-structural cracking.
(2) A novel CFRP optimization methodology: a multi-dimensional quantitative evaluation (radar-chart method) that integrates ultimate bearing capacity, crack suppression, material utilization, and relative interfacial slip, overcoming the reliance of conventional optimization on a single indicator such as the ultimate load capacity.
(3) Practical validation of a composite strengthening strategy: a complete closed-loop framework (inspection, diagnosis, simulation, remediation, and long-term monitoring) is established. Twelve-month field monitoring together with nonlinear FE analysis validates the effectiveness of the epoxy-grouting-plus-grid-CFRP system under residual shrinkage deformation. By limiting the CFRP effective strain to the debonding-controlled value specified in ACI 440.2R, the grid design is intended to promote a ductile failure mode in which steel yielding precedes interface debonding.

## 2. Project Overview and Characteristics of Roof Slab Cracks

The No. 2 workshop for high-end intelligent automobile components is a cast-in situ RC frame structure with two storeys above ground and partial five-storey zones. It measures 127.61 m transversely and 25.74 m longitudinally, with a total floor area of about 14,340 m^2^. More than three months after formwork removal of the second-floor slab, extensive cracks developed rapidly. The cracks were highly irregular; some ran through the full slab thickness and exposed the reinforcement, water seeped continuously from damp areas on the slab soffit, and white gelatinous deposits precipitated at the seepage locations. Drone imaging, supplemented by field reconnaissance, was used to map the macroscopic crack distribution. The survey revealed the following:

(1) The cracking occurred in autumn, when the diurnal temperature difference was about 12 °C.
(2) The cracked area accounted for about 70% of the floor area and continued to propagate outward.
(3) Most cracks ran diagonally on the slab surface and extended from the slab edges toward the interior.
(4) At the time of cracking, the slab carried only its self-weight and had not yet been subjected to service loads.

**Figure 1:**
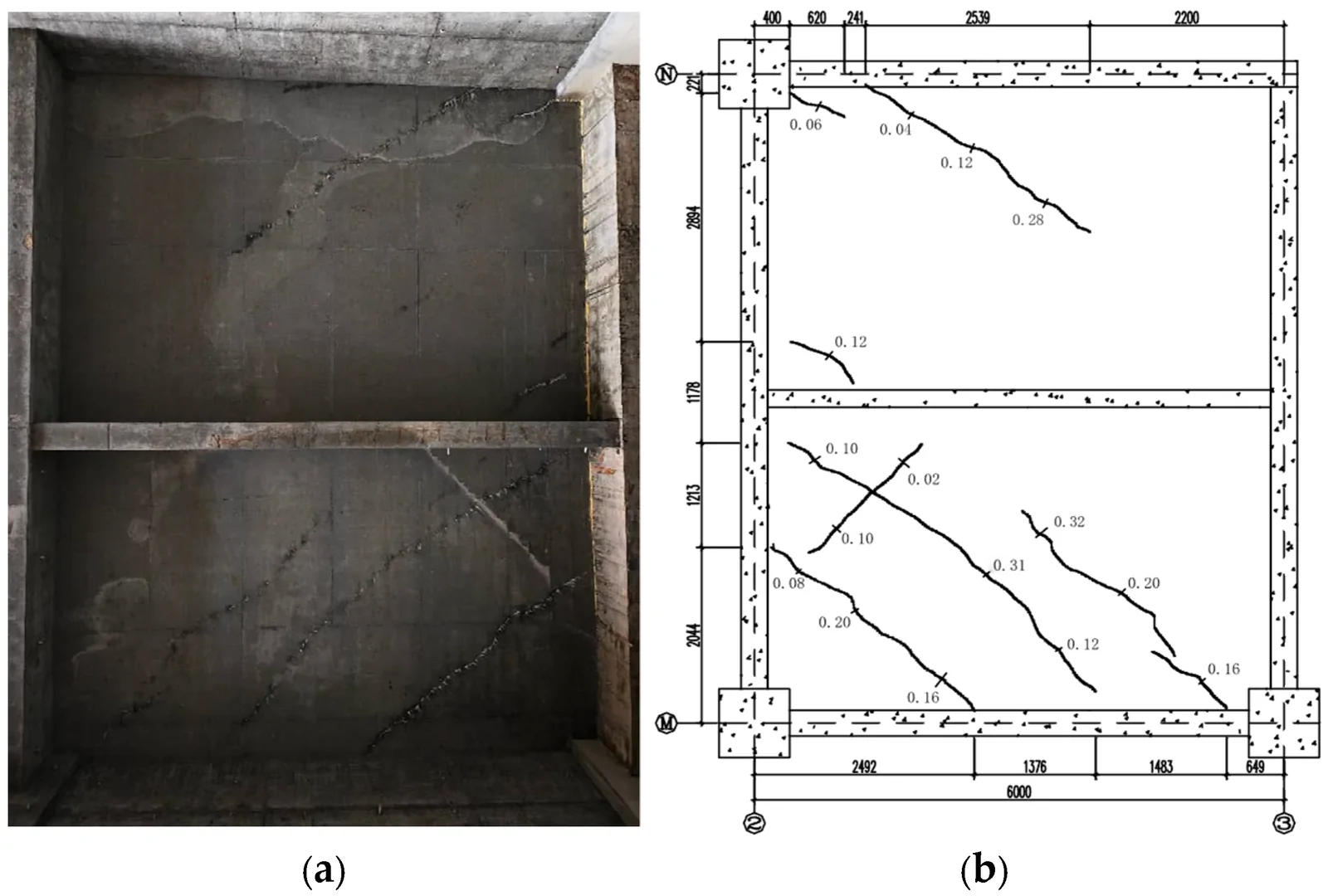
Cracking pattern of the roof slab between axes 2–3/M–N: (**a**) Field photographs; (**b**) Schematic diagram of cracks.

To determine the cracking cause, three representative panels (Slabs A, B, and C) were randomly selected for dedicated inspection, and their crack distribution, maximum crack width, estimated concrete strength, rebar spacing, and boundary conditions were compared quantitatively (Table 1). All three are continuous two-way slabs supported on primary and secondary beams along all four edges with identical boundary conditions, and their concrete-strength and rebar-spacing deviations are both within 5%. Slab A, on grid lines 2–3/M–N, exhibited the most extensive cracking and the largest maximum crack width (0.32 mm); it was therefore selected as the worst-case representative for the subsequent mechanism analysis and strengthening design, and the conclusions are representative of the other affected panels in the same batch. The crack morphology is shown in Figure 1a and the corresponding schematic in Figure 1b.

## 3. Testing Instruments and Analysis of Results

### 3.1. Testing Instruments

**Figure 2:**
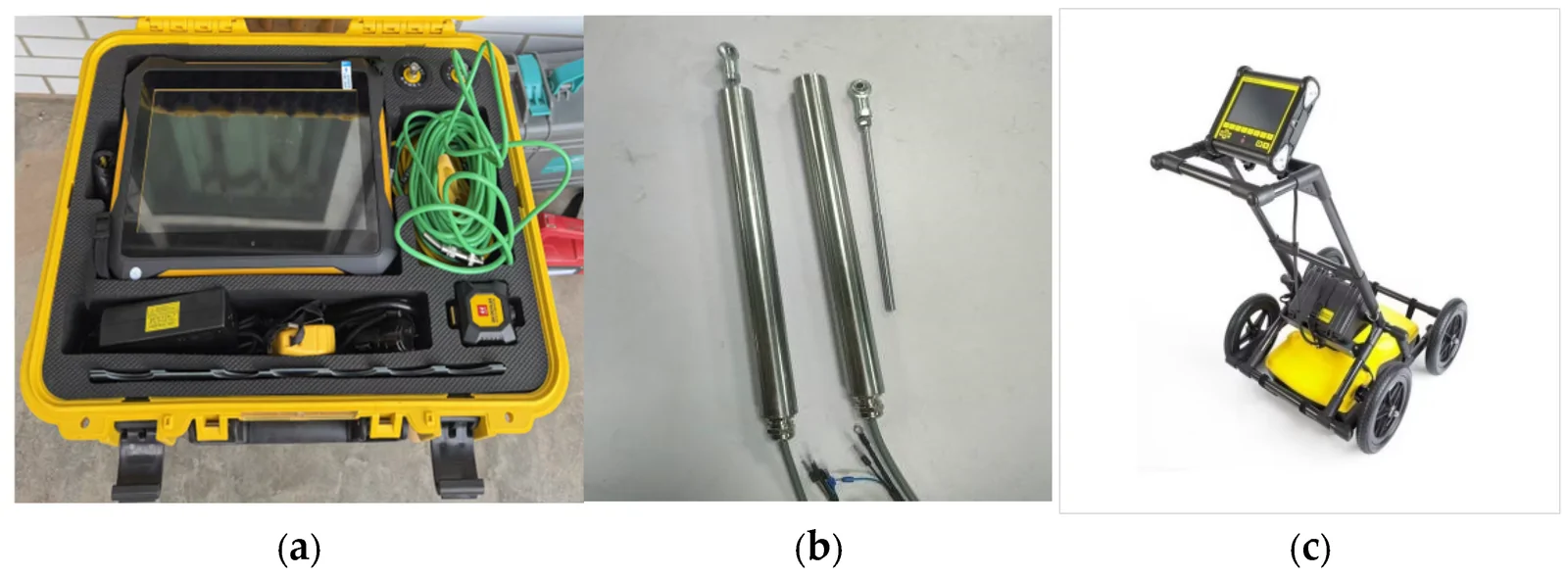
Partial field measurement instruments used in the test: (**a**) comprehensive crack tester; (**b**) displacement transducer; (**c**) ground penetrating radar (GPR).

To comprehensively characterize the slab defects, a digital rebound hammer, a rebar scanner, a slab-thickness gauge, an integrated crack detector, an electronic level, and ground-penetrating radar (GPR) were used for in situ testing. The rebound hammer (Jinan Langrui Testing Technology Co., Ltd., Jinan, China) estimated the concrete strength; the rebar scanner (Proceq, Schwerzenbach, Switzerland) provided the average rebar spacing and cover thickness; the slab-thickness gauge (Beijing Haichuang Gaoke Technology Co., Ltd., Beijing, China) measured the thickness at each point; the crack detector (Beijing Koncri Engineering Testing Technology Co., Ltd., Beijing, China) recorded the width and depth of surface cracks; the electronic level (Suzhou First Optical Instrument Co., Ltd., Suzhou, China) measured the relative vertical deformation at mid-span and supports; and GPR (Geophysical Survey Systems, Inc., Salem, NH, USA) detected hidden internal defects such as voids and delamination. The instruments are shown in Figure 2.

### 3.2. Analysis of Measurement Results

#### 3.2.1. Measurement Results of the Roof Slab

The designed thickness of the representative slab (axes 2–3/M–N) was 150 mm, and the measured thickness at five points ranged from 148 to 159 mm, averaging 154 mm (Table 2). The measured mid-span deformation was 2.23 mm. The cracks widened from the edges toward mid-span, and the maximum measured crack width reached 0.32 mm, exceeding the 0.30 mm limit [27].

The allowable deviations were ±10 mm for tensile-reinforcement spacing, −5 to +8 mm for cover thickness, and −5 to +10 mm for slab thickness [28]. As shown in Table 2, Table 3, Table 4 and Table 5, every measured index fell within the code tolerances, and the estimated concrete strength met the C30 design requirement, indicating that the construction quality and material performance complied with the code. Three powder samples were then scraped from the white deposits at seepage locations along the main cracks and prepared for X-ray diffraction (XRD) phase identification.

#### 3.2.2. Finite Element Model and Validation

Finite element models were built in ANSYS18.0. To ensure reproducibility, the detailed model parameters are given below.

(1) Element types and mesh: The concrete was modeled with eight-node Solid65 elements; the longitudinal reinforcement was modeled discretely with Link180 truss elements; the externally bonded CFRP sheets were modeled with Shell181 shell elements; and the CFRP–concrete interface was represented by COMBIN39 nonlinear spring elements. A mapped mesh was used, with a global in-plane element size of 100 mm and five elements through the slab thickness.
(2) Material constitutive models and elastic properties:

Concrete: A multi-linear kinematic hardening model was adopted, while for tension, the maximum principal tensile stress criterion with post-cracking stiffness degradation (tension softening) was used. The concrete parameters were taken as *Ec* = 30 GPa, *νc* = 0.2, and *ft* = 2.70 MPa. The steel rebars were modeled as an ideal elastoplastic material with *Es* = 200 GPa and *fy* = 400 MPa. According to the field inspection, the slab reinforcement comprised bars of different diameters and spacings. For each bar type, the nominal cross-sectional area of a single bar was calculated as *As* = *π*d^2^/4 and input as the section property of the corresponding rebar elements.

CFRP sheets: A linear elastic orthotropic model was adopted, with *Ef* = 230 GPa, ultimate tensile strength *ffu* = 3400 MPa, and thickness *tf* = 0.167 mm.

Interface: The bilinear bond–slip model of Lu et al. [29] was applied to the COMBIN39 springs, with the initial stiffness *K*0 = 4.0 MPa/mm, peak bond stress *τmax* = 2.5 MPa, and fracture energy *Gf* = 0.5 N/mm defined through the force–displacement real constants.

(3) Boundary conditions and loading: the bottom nodes of the surrounding columns were fully fixed (all translational and rotational degrees of freedom restrained). Loading was applied in two steps: self-weight first, followed by a uniform live load of 4.0 kN/m^2^ on the slab surface.
(4) Convergence and failure criteria: A force-based convergence criterion with a tolerance of 0.05 was adopted. Failure was defined as (a) concrete cracking, (b) yielding of the tensile reinforcement, or (c) CFRP rupture or debonding.

**Figure 3:**
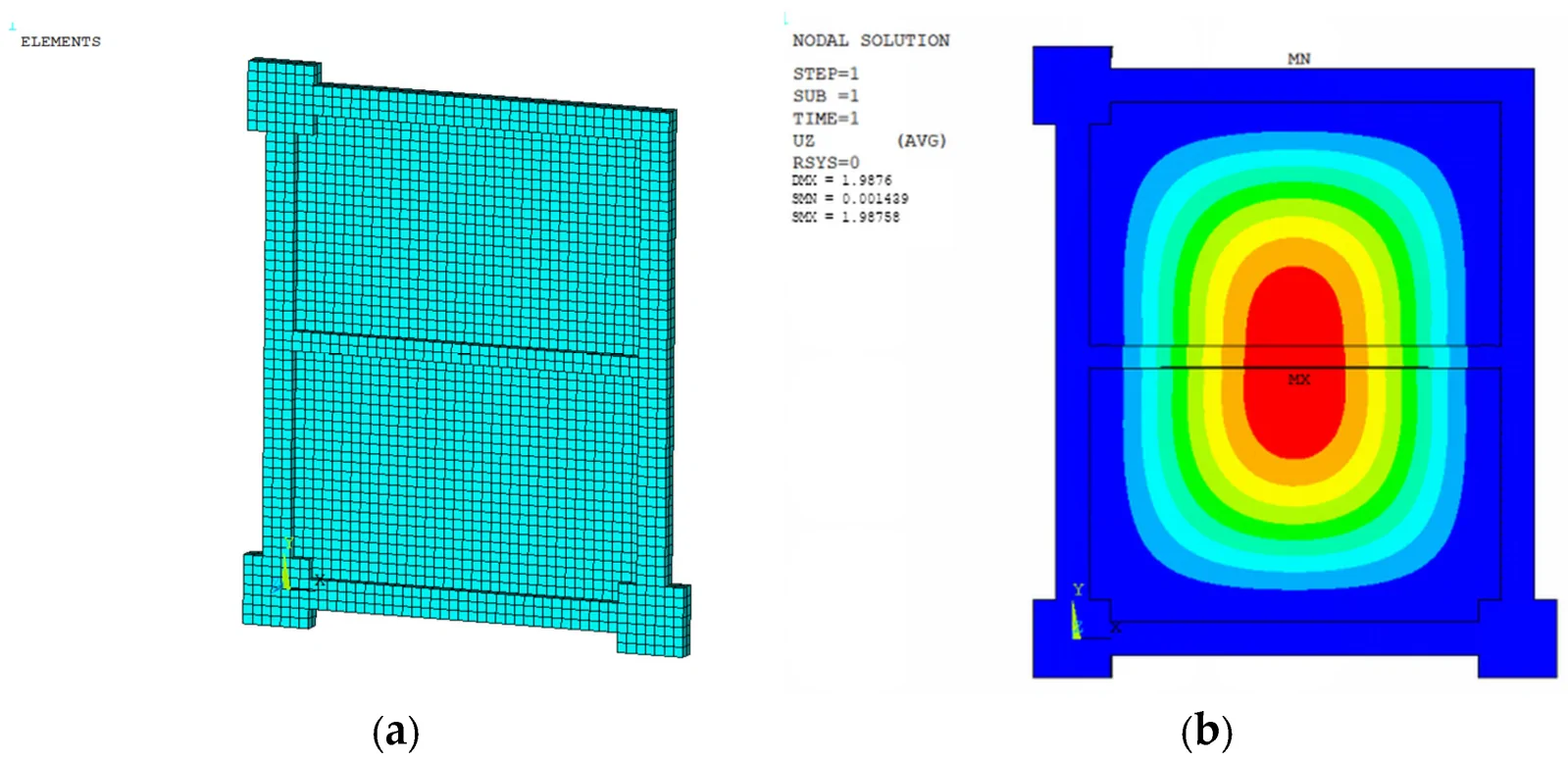
ANSYS18.0 finite element simulation diagram: (**a**) finite element model of the actually measured roof slab between Axes 2~3/M~N; (**b**) displacement and stress contours of the slab under self-weight load. (Note: The color gradient from blue to red represents the increase in displacement/stress).

The resulting FE model of the representative slab is shown in Figure 3a. Self-weight and the uniform live load were applied separately to obtain the mid-span deflections, and the displacement and stress contours under self-weight are shown in Figure 3b.

Model validation. The FE-calculated self-weight deflection was 1.98 mm, which is 11.2% below the measured value of 2.23 mm. Rather than attributing this difference solely to field measurement disturbance or temperature, a sensitivity analysis was performed on the concrete elastic modulus and the support rotational stiffness. In the pre-cracking linear elastic regime, the slab deflection scales inversely with the effective stiffness, *w* ∝ 1/*Ec*; reducing *Ec* by 10% (from 30 to 27 GPa), to reflect the mix-proportion defects and early-age loading, raises the predicted deflection to about 2.20 mm and reduces the deviation to 1.3%. Independently releasing the support rotational restraint by 20% to allow for micro-rotation of the monolithic beam–slab joints acts in the same direction and produces a smaller, secondary increase. Both effects are physically plausible, whereas the resolution of the electronic level (about 0.1 mm) accounts for only a minor part of the 0.25 mm gap. The discrepancy therefore stems mainly from conservative stiffness and boundary idealizations rather than measurement error, which validates the FE model for predicting the overall macroscopic response.

For a general instrument-and-meter workshop, the floor live load during normal service was taken as 4.0 kN/m^2^ [30]. The mid-span deflections under the two loading conditions are summarized in Table 6. Under self-weight, the measured and simulated deflections are 2.23 and 1.98 mm, respectively; applying the 4.0 kN/m^2^ live load increases the simulated deflection to 4.89 mm. Even so, the maximum deflection remains well below the code limit of 24.20 mm, confirming that the flexural performance fully satisfies the serviceability requirements.

## 4. Analysis of the Roof Slab Cracking Mechanism

The concrete strength, reinforcement layout, cover thickness, slab thickness, and mid-span deflection were integrated, and the flexural bearing capacity was rechecked. Structural cracking was thereby preliminarily excluded, and the non-structural causes were then identified.

**Figure 4:**
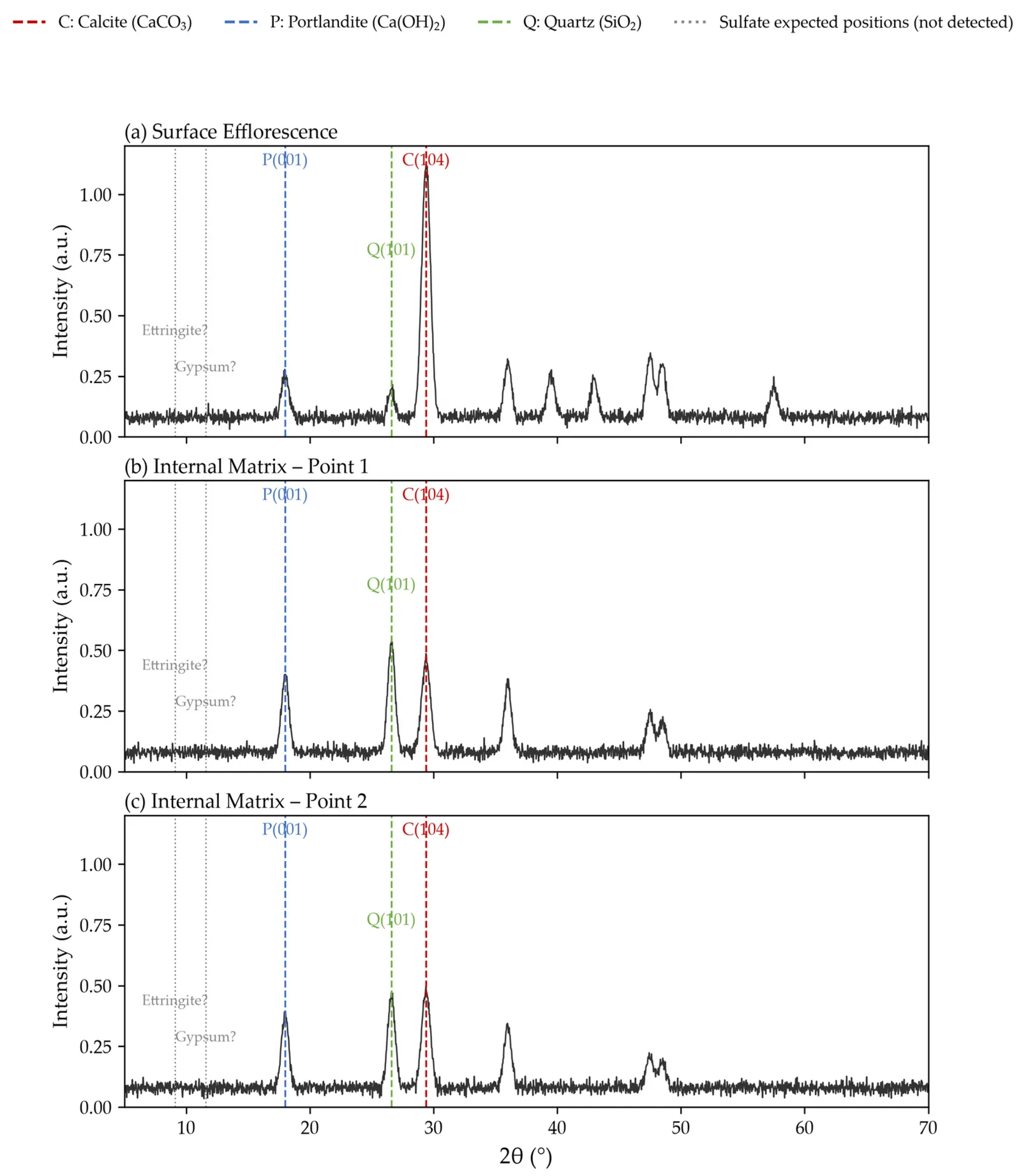
XRD patterns of concrete samples.

The XRD patterns (Figure 4) show that the surface efflorescence consists mainly of calcite (C, CaCO_3_), with minor portlandite (P, Ca(OH)_2_) and a trace of quartz (Q, SiO_2_); the trace quartz is attributable to fine sand or airborne dust adhering to the surface. This assemblage is characteristic of secondary efflorescence formed by carbonation of leached calcium hydroxide. The internal matrix samples display the same crystalline assemblage—calcite, portlandite, and quartz—where quartz originates from the siliceous fine aggregate. At the characteristic positions of crystalline sulfate-bearing phases, namely ettringite (2θ ≈ 9.1°) and gypsum (2θ ≈ 11.7°), all three patterns show only flat background noise, with no detectable peaks above the instrument’s 1–2 wt.% detection limit; hence, no abnormally enriched crystalline ettringite or gypsum indicative of sulfate attack (or delayed ettringite formation) is observed. Because XRD identifies only crystalline phases above this limit and cannot detect amorphous products such as alkali–silica gel, this result specifically rules out crystalline sulfate-type expansion rather than every possible chemical action. Combined with the crack morphology (diagonal cracks propagating from the edges toward the interior under self-weight) and the confirmed mix-proportion defects, the evidence supports restrained drying shrinkage as the dominant cause of the extensive cracking, rather than chemical corrosion.

To examine the material factors, the original design mix was reviewed, and the retained specimens were sampled and tested. The comparison (Table 7) reveals severe defects in the as-placed concrete. The actual water-to-binder (W/B) ratio reached 0.55, the upper limit for exposure class XC3 in EN 206 [31]; the cement content was 300 kg/m^3^, barely meeting the EN 206 minimum [31]; the fly-ash content was as high as 25%, the EN 450-1 upper limit [32]; and the mud content of the fine aggregate reached 4.5%, well above the 3.0% limit in ASTM C33 [33]. These defects markedly increase the drying-shrinkage deformation and provide the material basis for the subsequent shrinkage cracking.

To quantify the shrinkage stress induced by the problematic materials, the calculation was based on the internationally recognized theoretical framework for restrained shrinkage. First, the ACI 209R-92 model [34] was adopted to predict the drying shrinkage strain. Considering the actual non-standard conditions of this project (relatively high water-cement ratio, excessive fly ash content, and high silt content in fine aggregate), a comprehensive correction coefficient $\prod_{i=1}^{n}M_{i}$ was introduced to modify the ultimate shrinkage strain. The drying shrinkage strain at age *t* is expressed as

(1) $$\varepsilon_{\mathrm{sh}}\left(t\right)=3.24\times 10^{-4}\cdot \left(1-e^{-\mathrm{bt}}\right)\prod_{i=1}^{10}M_{i}$$

where *b* is the shrinkage rate coefficient, taken as 0.01 under conventional curing; *t* is the concrete age, taken as 90 days; *M*_1_~*M*_10_ are modification coefficients. Based on the material defects, the comprehensive correction coefficient is taken as $\prod_{i=1}^{10}M_{i}$ = 1.75 [34]. Substituting these values into Equation (1) yields

*ε_sh_*(90) = 3.365 × 10^−4^, (2)

For the multi-span cast-in situ frame, the beams are restrained by columns and their horizontal displacement is essentially locked, so the rigid-boundary assumption (zero support displacement) was adopted, and the free shrinkage strain of the slab was used directly as the driving deformation for restraint stress [34,35]. The restraint characteristic coefficient *β* and the constraint-effect coefficient *X* were calculated as

(3) $$\beta =\sqrt{\frac{C_{x}}{h\cdot E\left(t\right)}}=5.352\times 10^{-4}{\mathrm{mm}}^{-1},$$

(4) $$X=1-\frac{1}{\cosh\left(\mathrm{\beta L}/2\right)}=0.6188,$$

where *Cx* is the horizontal resistance coefficient, taken as 1.5 N/mm^3^ for cast-in situ beam–slab systems under moderate-to-strong restraint [34], *h =* 154 mm is the measured slab thickness, and *E*(*t*) is the elastic modulus of concrete at age *t*. In this study, the 90-day elastic modulus was taken as 3.4 × 10^4^ based on the measured rebound strength, and L = 6050 mm is the effective slab length.

To account for the stress relaxation caused by tensile creep, a reduction function *H*(*t*) was introduced. Its value was supported by Altoubat and Lange [36], who found that tensile creep relaxes early-age restrained tensile stress by about 50%. The maximum restrained shrinkage stress *σmax* was then calculated as

*σ_max_* = *E*(*t*) ⋅ *ε_sh_*(90) ⋅ *H*(*t*) ⋅ *X* = 3.19 MPa, (5)

where *H*(*t*) is the reduction function for stress relaxation, conservatively taken as 0.45.

**Figure 5:**
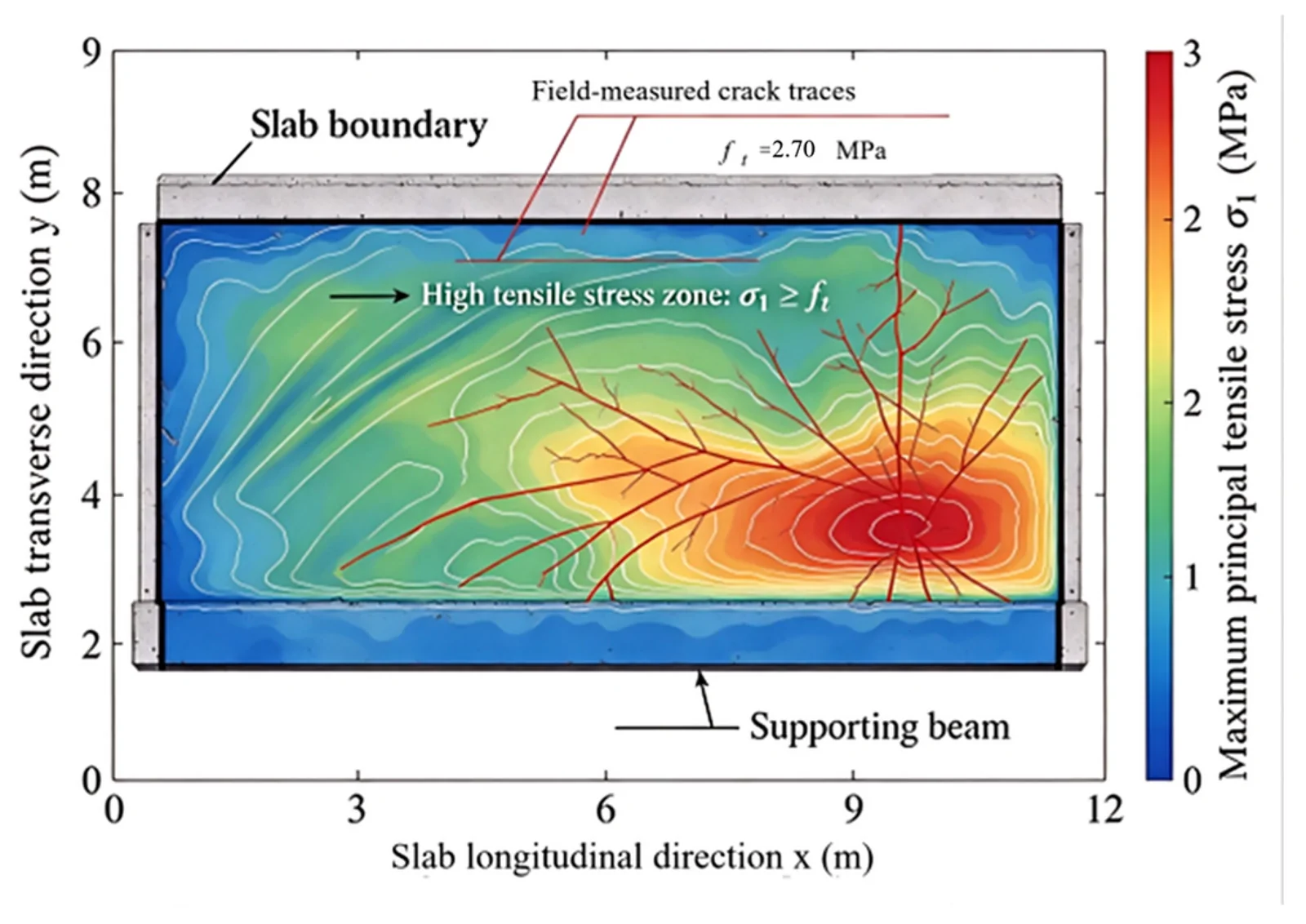
Contour of maximum principal tensile stress and superimposed field crack traces.

As shown in Figure 5, the contour of the maximum principal tensile stress (*σ*1) exhibits a distinct high-stress zone near the mid-right area of the slab. The superimposed field crack traces align closely with this zone and radiate outward from the stress core. The peak restrained shrinkage stress reaches 3.19 MPa, exceeding the characteristic axial tensile strength (5% fractile, f_ctk,0.05_) of C45 concrete (2.70 MPa) [27], which satisfies the macroscopic mechanical condition for crack initiation.

**Figure 6:**
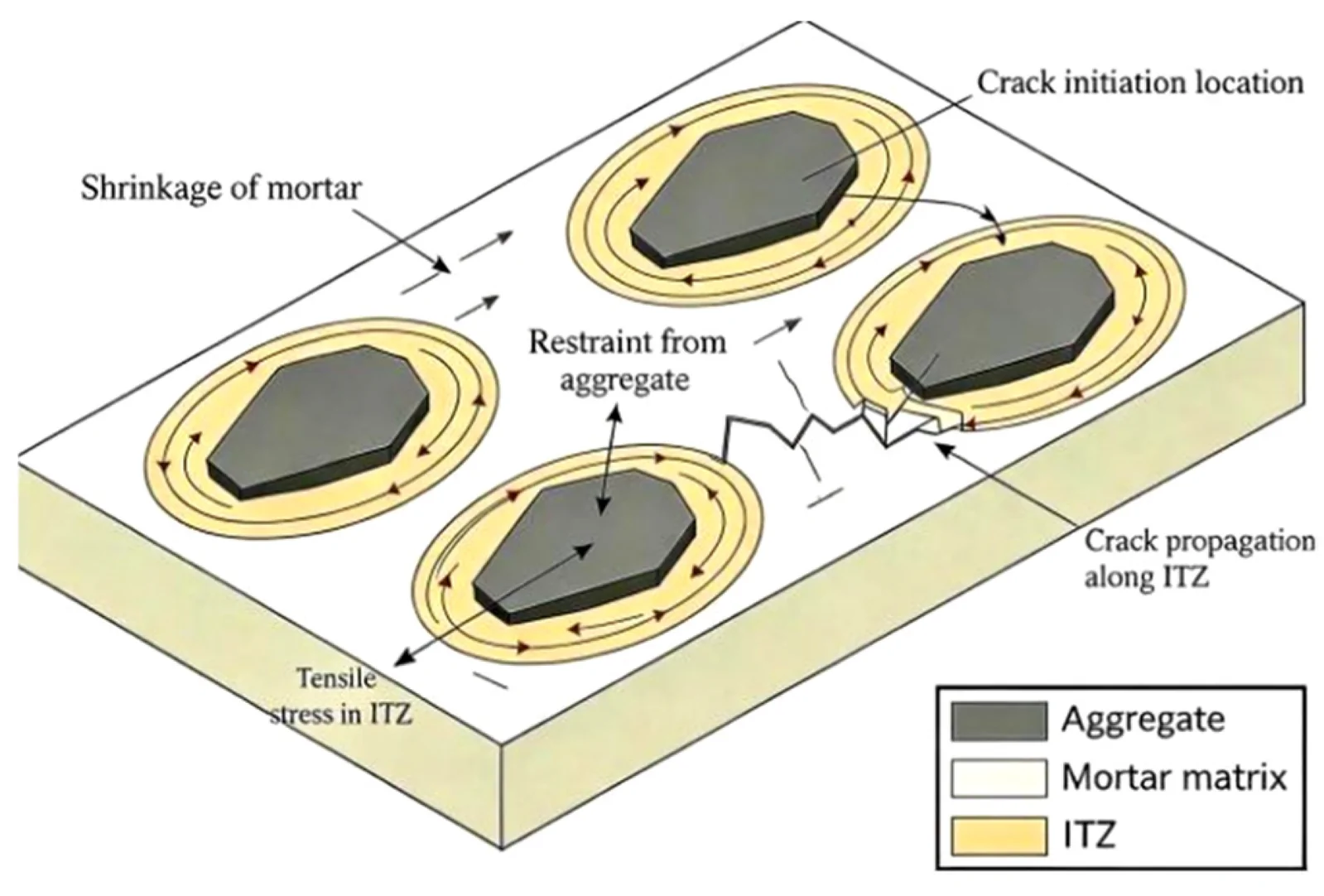
Cracking mechanism diagram of concrete aggregate–mortar ITZ.

At the mesoscale, concrete is a three-phase composite of coarse aggregate, mortar matrix, and ITZ (Figure 6). The ITZ is porous and has poor tensile resistance. Reported ratios of ITZ-to-mortar tensile strength range from about 33% to 67%, depending on the aggregate mineralogy, water-to-cement ratio, and curing conditions [37,38]. For a mortar tensile strength of about 3.4 MPa [39,40], these ratios give ITZ tensile strengths of about 1.11–2.28 MPa; Notably, this upper bound (2.28 MPa) is substantially lower than both the characteristic axial tensile strength (5% fractile, f_ctk,0.05_) of C45 concrete (2.70 MPa) and the calculated maximum shrinkage stress (3.19 MPa). Therefore, microcracks inevitably initiate preferentially within the weak ITZ prior to macroscopic cracking. The unreasonable mix proportions increase the drying shrinkage, making the ITZ the weakest link under shrinkage-induced tension. Under the restraint of the beams and columns, as shrinkage tensile stress develops in the slab and exceeds the ITZ tensile strength, microcracks propagate along these weak interfaces and coalesce into macroscopic cracks. This progressive damage mechanism perfectly explains why the field cracks in Figure 5 are densely distributed and radiate from the high-stress zone.

## 5. Comparison and Verification of CFRP Strengthening Schemes

### 5.1. Design of CFRP Strengthening Schemes

To shorten the repair period and reduce cost, externally bonded CFRP sheets were adopted, and a strengthened FE model was built on the basis of the slab model in Figure 3a. The element types and the CFRP–concrete interface model described in Section 3.2.2 (Shell181 sheets and COMBIN39 springs) were retained.

The CFRP parameters followed the ACI 440.2R-23 guideline [41] (*Ef* = 230 GPa, *ffu* = 3400 MPa, *tf* = 0.167 mm; ultimate rupture strain *εfu* = 0.0148). The effective strain *εfe* was taken as the smaller of the rupture strain and the debonding-controlled strain, in accordance with the guideline, to ensure the debonding safety margin.

**Figure 7:**
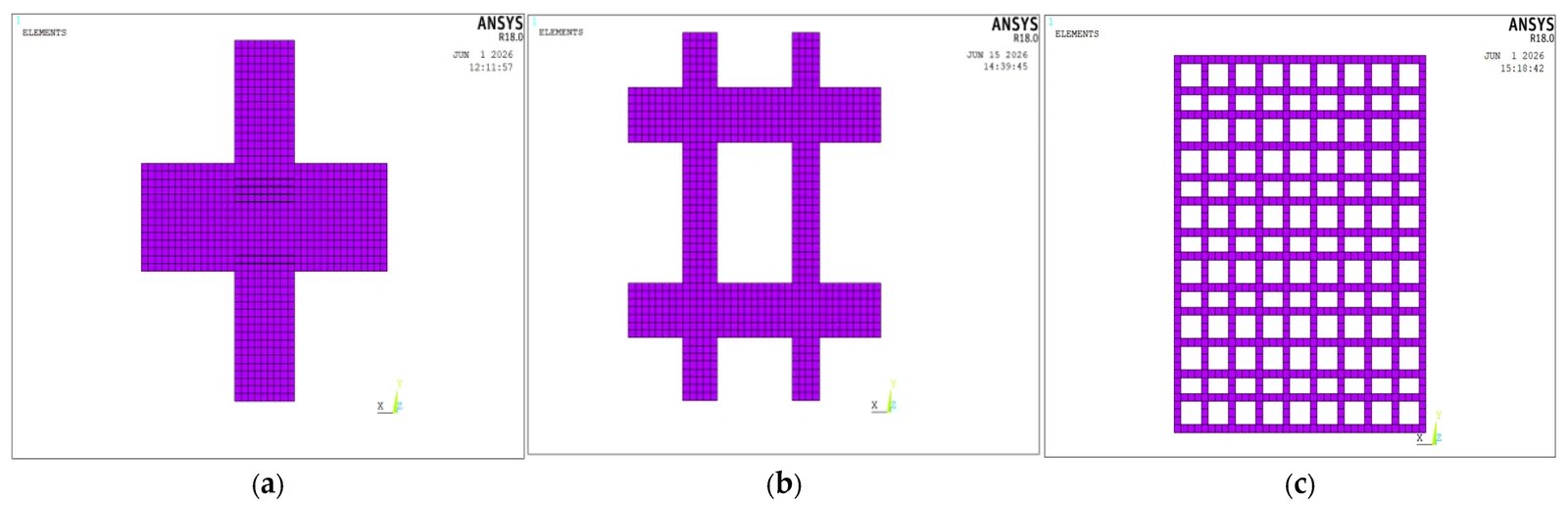
CFRP sheet strengthening scheme: (**a**) cross-shaped layout; (**b**) hash-shaped layout; (**c**) grid-shaped layout.

The force–displacement law of the springs was the bilinear bond–slip model of Lu et al. [29]. With the same total CFRP consumption, three layouts were designed—cross-shaped, hash-shaped, and grid-shaped—and bonded to the slab bottom (Figure 7); the CFRP sheet density was identical in all three, and the specifications are given in Table 8.

### 5.2. Comparison and Optimization of CFRP Strengthening Schemes

#### 5.2.1. Comparative Analysis of Strengthening Schemes

**Figure 8:**
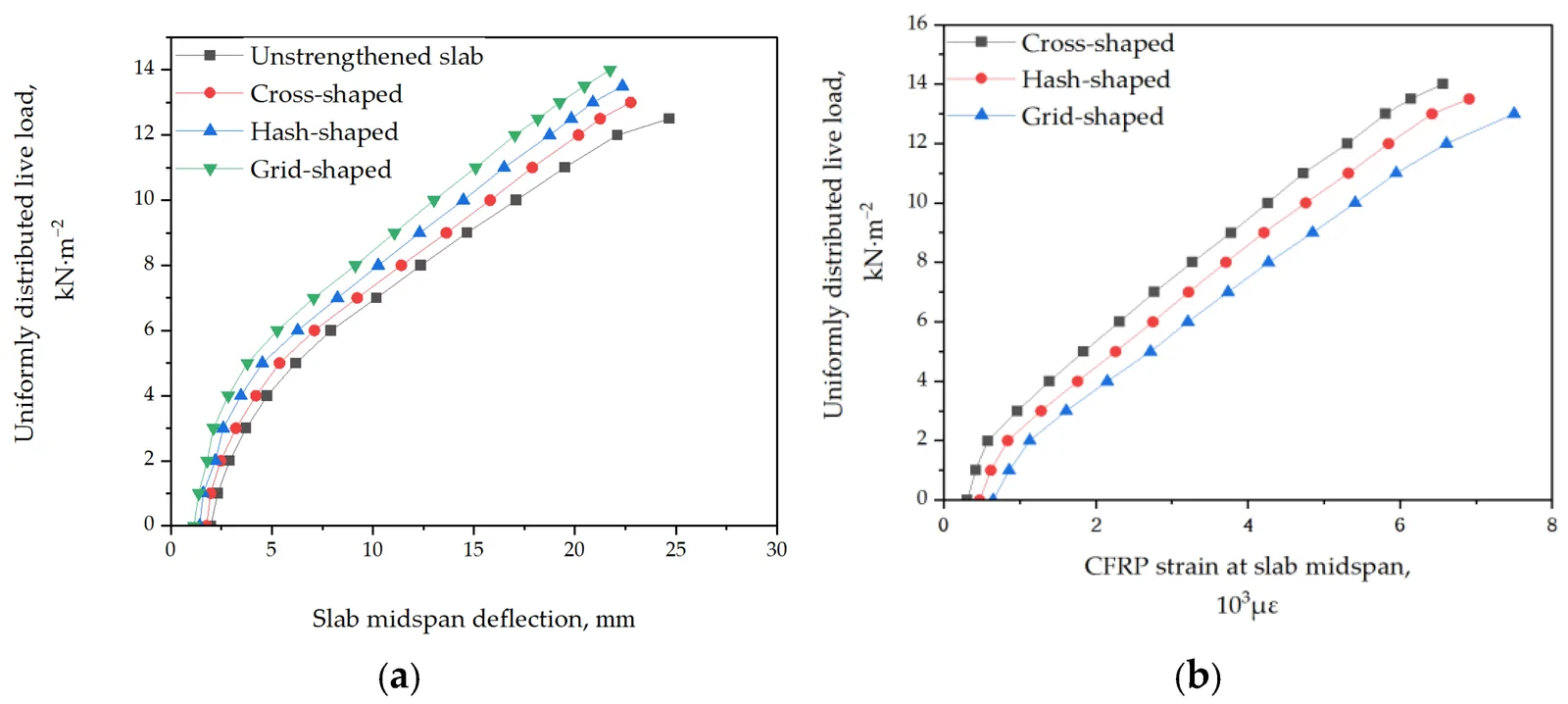
Finite element simulation results for different strengthening schemes: (**a**) variation in midspan deflection with uniformly distributed live load; (**b**) variation in CFRP strain with uniformly distributed live load.

Self-weight and the uniform live load were applied sequentially, and the variations in the mid-span deflection and CFRP strain with the load were obtained for each scheme (Figure 8).

The CFRP layout markedly changes the stress transfer, crack restraint, and interfacial stress state of the two-way slab [42,43,44]. At the same load level, the mid-span deflection ranked as unstrengthened > cross-shaped > hash-shaped > grid-shaped (Figure 8a), indicating that the CFRP sheets increased the flexural stiffness. The cross-shaped scheme concentrates the CFRP at mid-span with limited coverage, whereas the bidirectional orthogonal grid provides the most uniform restraint on flexural deformation.

At the same load level, the mid-span CFRP strain ranked as cross-shaped > hash-shaped > grid-shaped (Figure 8b). The cross-shaped layout showed marked stress concentration and a high debonding risk; the grid layout distributed the stress uniformly, mitigating the concentration and the debonding risk; and the hash-shaped layout was intermediate.

#### 5.2.2. Four-Dimensional Evaluation and Scheme Optimization

To evaluate the three configurations comprehensively, four indicators were used: ultimate bearing capacity, CFRP material utilization, crack suppression ratio, and relative interfacial slip. They are defined as follows:

(1) Ultimate bearing capacity: Obtained directly from the ANSYS18.0 model under incremental loading.
(2) CFRP material utilization ratio: (maximum tensile stress in the CFRP/ultimate tensile strength of the CFRP) × 100%.
(3) Crack suppression ratio: Because Solid65 cannot output continuous crack widths directly, the tensile steel stresses before and after strengthening were extracted with APDL, and the predicted crack width *Wpred* was back-calculated from the Eurocode 2 (EN 1992-1-1) crack-width formula [27]. The suppression ratio is *ηcr* = (*Wpred*,*unstr* − *Wpred*,*str*)/*Wpred*,*unstr* × 100%. This is a relative index derived from FE results and code formulas, used only to compare schemes; it is not an absolute field-measured crack width.
(4) Relative interfacial slip: The interfacial slip *s* was taken as the relative longitudinal displacement between the CFRP and concrete nodes connected by the COMBIN39 springs, and the interfacial shear stress was obtained by dividing the spring force by the bonded area. The safety reserve was assessed by comparing the maximum slip under static loading with the ultimate slip threshold *su* = 0.4 mm. This index reflects only short-term static slip resistance; because creep, fatigue, moisture, temperature, and adhesive aging were not modeled, it does not represent long-term interfacial durability.

The bearing capacity, material utilization, and crack suppression ratio were treated as positive indicators using Equation (6), whereas the relative interfacial slip was treated as a negative indicator using Equation (7).

Positive indicator:

(6) $$S\;=\;1\;+\;9\times \frac{X_{i}-X_{\min}}{X_{\max}-X_{\min}}$$

**Figure 9:**
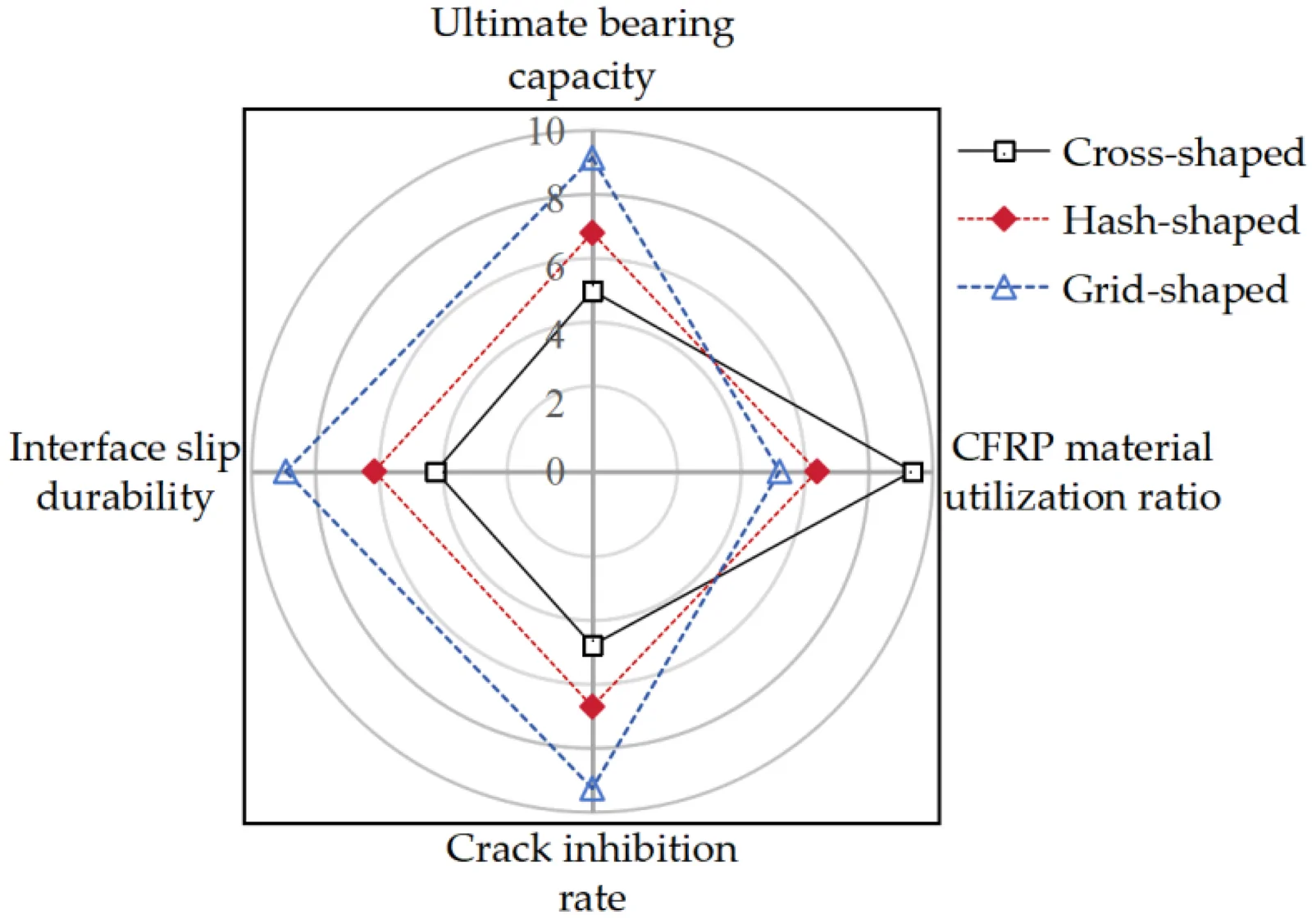
Four-dimensional evaluation radar chart for CFRP sheet strengthening.

Negative indicator:

(7) $$S\;=\;1\;+\;9\text{ × }\frac{X_{\max}-X_{i}}{X_{\max}-X_{\min}}$$

where *S* is the evaluation score; *X_i_* is the finite element calculated value of the case; *X*_max_ and *X*_min_ are the maximum and minimum values of the index in this group. The resulting standardized scores for the three schemes are listed in Table 9; the corresponding radar chart is shown in Figure 9.

As shown in Figure 9 and Table 9, the cross-shaped scheme was superior only in material utilization and deficient in the other three dimensions. In contrast, the grid scheme performed most evenly across all dimensions; at the cost of a slightly lower material utilization, it achieved the best bearing capacity, crack control, and interfacial slip resistance, and it is therefore the most suitable scheme for the cracked slab.

**Figure 10:**
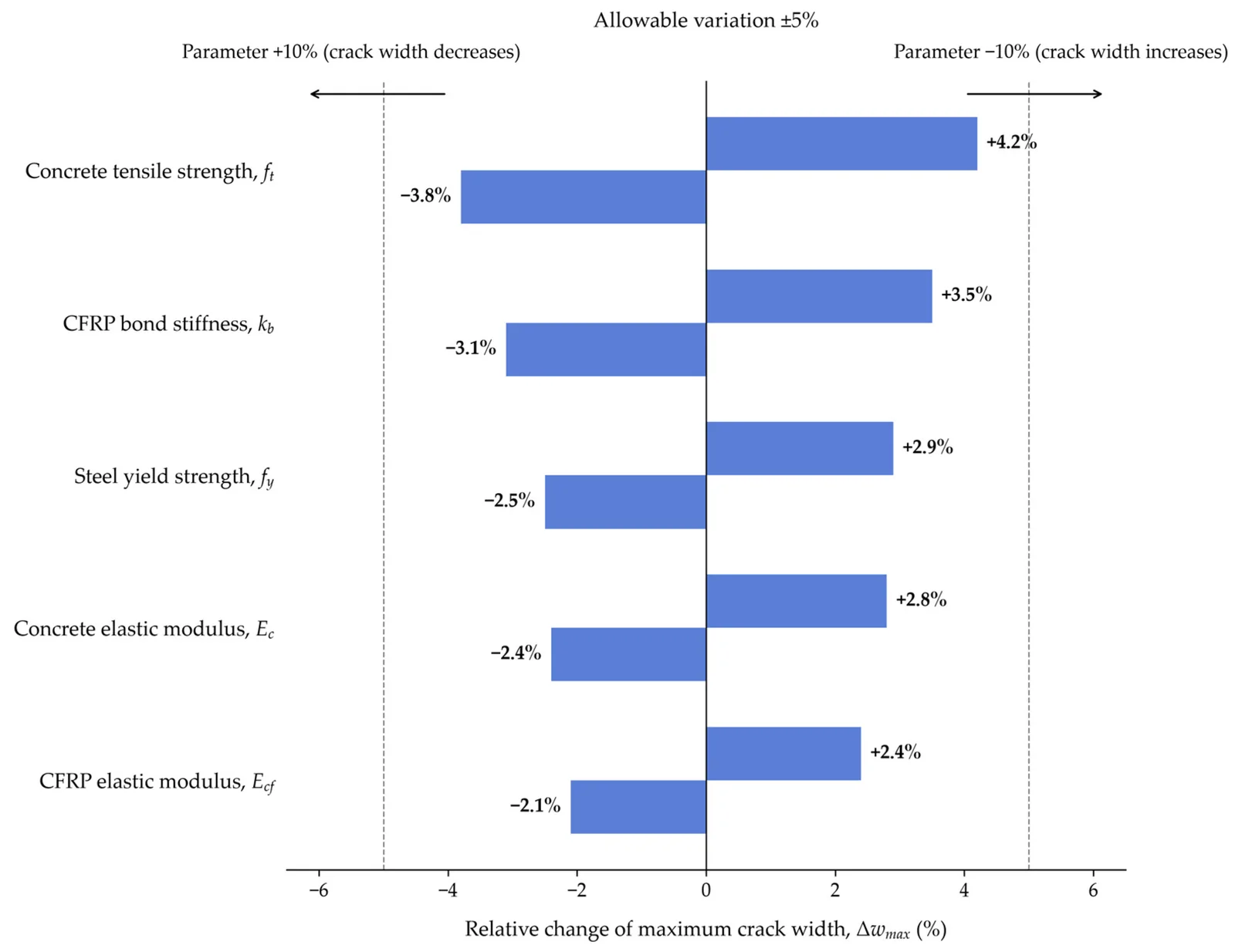
Sensitivity of maximum crack width to 10% variation in key material parameters.

To verify the optimal scheme under material uncertainty, a sensitivity analysis was performed with a ±10% variation in the concrete tensile strength (*ft*), CFRP bond stiffness (*kb*), steel yield strength (*fy*), concrete elastic modulus (*Ec*), and CFRP elastic modulus (*Ef*), as shown in the tornado diagram (Figure 10). The crack width was most sensitive to *ft* and *kb*: a 10% decrease increased the crack width by up to 4.2% and 3.5%, respectively, while a 10% increase reduced it by up to 3.8% and 3.1%. All changes remained within the ±5% bounds (dashed lines in Figure 10), demonstrating the robustness of the grid scheme to realistic material uncertainty.

### 5.3. CFRP Strengthening Technology and Effect Inspection

**Figure 11:**
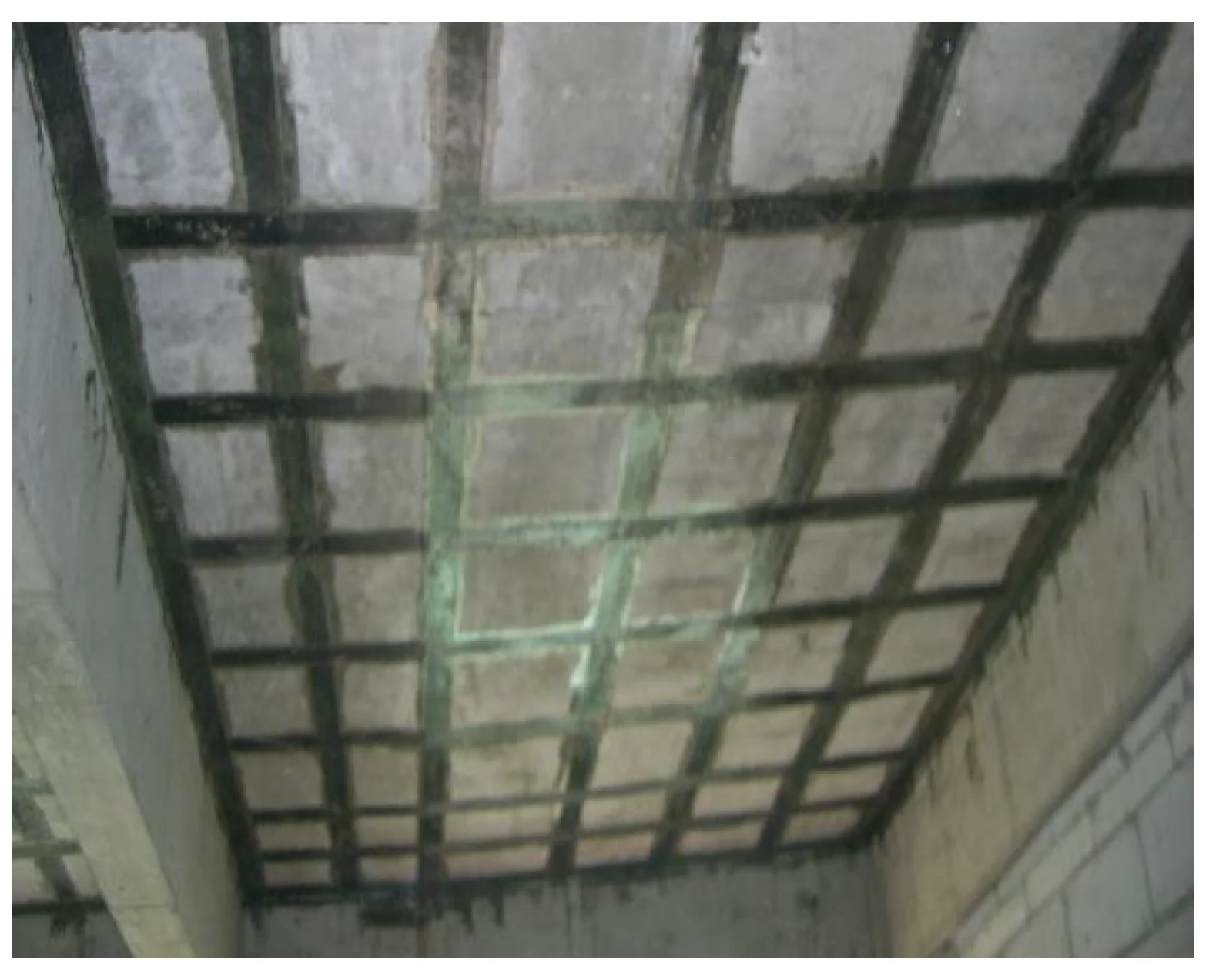
Grid-shaped CFRP Strengthening of Roof Slab between Axes 2~3/M~N (partial view).

**Figure 12:**
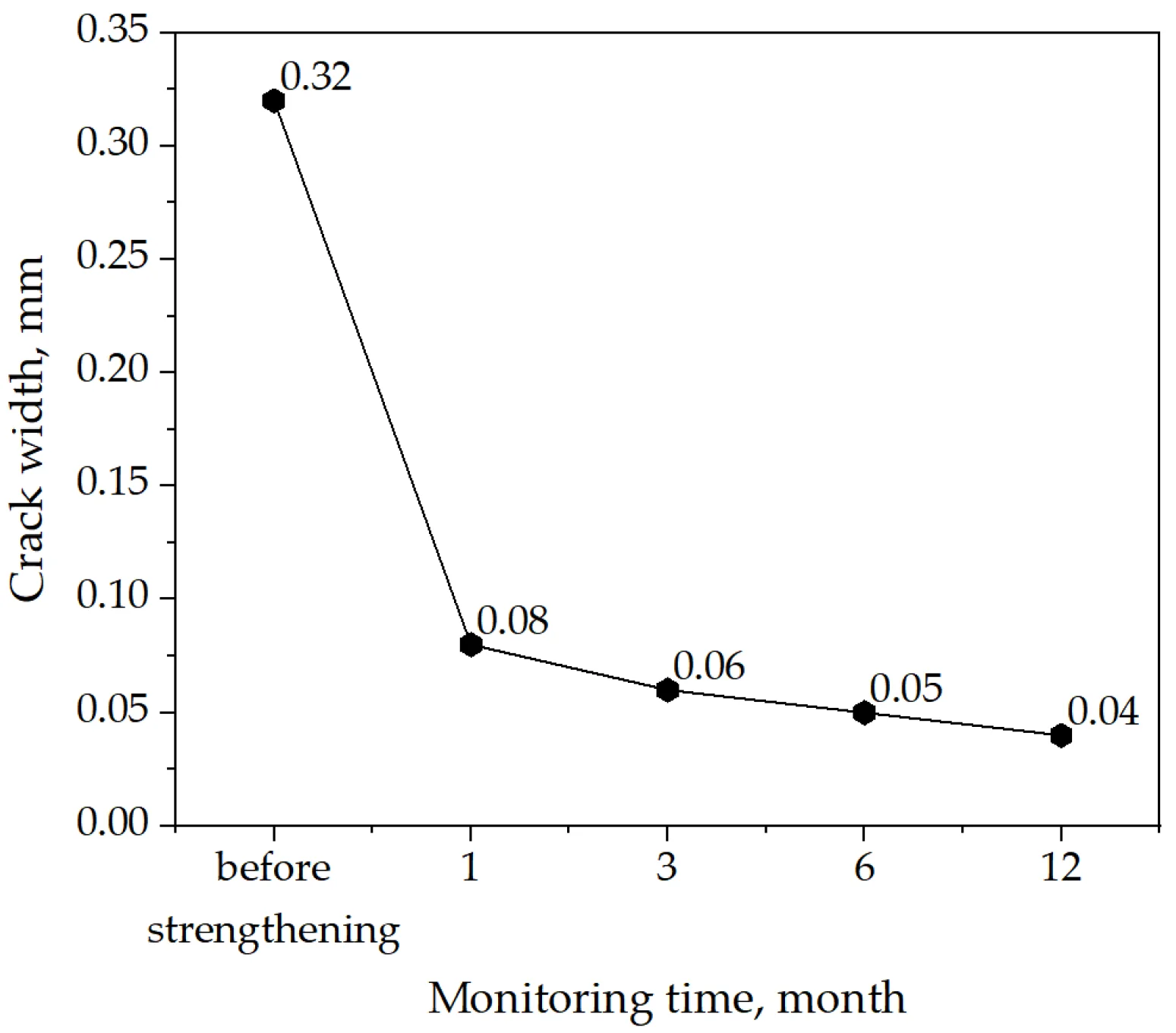
Variation curve of the crack width of the roof slab.

A combined scheme of low-pressure crack grouting and externally bonded grid-shaped CFRP was adopted (Figure 11 and Figure 12). The grouting filled the internal voids of the cracks, while the grid CFRP enhanced the restraint of the slab tension zone and arrested crack propagation.

1. CFRP Strengthening Technology

Before grouting, nozzles were installed at intervals along the cracks, and the crack surfaces were sealed. Epoxy resin was injected at 0.2–0.4 MPa, with the volume and pressure controlled to avoid disturbing the slab. Before bonding the CFRP, the concrete surface was cleaned and leveled, primer and impregnating resin were applied, and the grid carbon-fiber sheets were laid with a lap length of at least 150 mm and rolled repeatedly to ensure intimate contact with the substrate.

2. Strengthening Effect Inspection

After strengthening, the slab was monitored for about 12 months. The maximum crack width decreased from 0.32 mm before strengthening to 0.08 mm at 1 month, 0.06 mm at 3 months, 0.05 mm at 6 months, and 0.04 mm at 12 months. Closure occurred mainly within the first three months and then stabilized, and no propagation or new cracks appeared under seasonal temperature changes. The CFRP sheets were laid to the edges of the supporting beams, which provided the boundary restraint, and no U-shaped anchor strips were used at the rib beams; no end debonding occurred during the one-year monitoring.

## 6. Conclusions

Based on field inspection, XRD analysis, analytical calculations, FE simulation, and 12-month monitoring, this study investigated the cracking mechanism of an industrial roof slab and optimized the CFRP strengthening layout. The main conclusions are as follows.

(1) Cracking mechanism and material influences: Structural overloading and crystalline sulfate-type chemical attack were reasonably excluded by field testing and XRD analysis. The extensive cracking was driven mainly by restrained drying shrinkage, aggravated by severe mix-proportion defects (a high W/B ratio, excessive fly ash, and a high mud content). The preferential initiation of microcracks in the ITZ is presented as a mechanically inferred mechanism based on stress calculations and the literature, because direct SEM observation was not performed.
(2) Novel optimization methodology and robustness: Under identical total CFRP consumption, the multi-dimensional radar-chart evaluation showed that the orthogonal grid layout outperformed the cross- and hash-shaped layouts in bearing capacity, crack suppression, and interfacial slip resistance. A ±10% sensitivity analysis kept all crack-width variations within ±5%, confirming the robustness of the grid scheme to material uncertainty.
(3) Practical validation and failure control: The combined low-pressure grouting and grid-CFRP strategy reduced the maximum crack width from 0.32 to 0.04 mm after 12 months, and the structural performance remained stable. This outcome is attributed to the synergistic action of the grouting and CFRP system rather than CFRP alone. Because the CFRP effective strain was limited to the debonding-controlled value specified in ACI 440.2R, the grid scheme is expected to favor a ductile failure mode with steel yielding preceding interface debonding rather than premature debonding.
(4) Applicability scope: The grid-strengthening recommendation applies strictly to cracked two-way slabs with comparable span, thickness, reinforcement ratio, and load level. For slabs with larger spans or heavier loads, additional end anchorage (e.g., U-jackets) is recommended to prevent premature debonding.

## 7. Limitations

Despite these insights, several issues remain open because of the constraints of practical engineering:

(1) Microstructural characterization: The framework focuses on macroscopic stress–strain behavior and overall crack propagation, so the preferential ITZ initiation is presented as a mechanically inferred mechanism based on stress calculations and the literature. Direct microstructural verification (e.g., SEM) was beyond the scope of this case study and is a priority for future work.
(2) Boundary simplification: The FE model adopted fully fixed supports. The accompanying sensitivity analysis showed that the effective concrete modulus dominates the validation discrepancy (a 10% reduction restores the deviation to 1.3%), whereas the support rotational restraint was varied only over a limited range. Future work should incorporate explicit semi-rigid boundary models with rotational springs to capture local joint deformation more accurately.
(3) Interfacial parameters: The CFRP–concrete bond–slip law was adopted from the published model of Lu et al. rather than calibrated with local double-shear tests. Although the sensitivity analysis showed that the optimization ranking is robust to these parameters, pull-off tests would provide more accurate absolute slip values.
(4) Long-term performance and extreme environments: only the short-term static response and the initial crack closure were examined, and creep, fatigue, moisture cycles, and fire resistance were not modeled. The 12-month monitoring is a preliminary validation; longer monitoring and a fireproofing assessment are needed to evaluate the service life.
(5) Evaluation indices and optimization scope: The crack suppression ratio is a relative index derived from FE results and code formulas rather than an absolute measurement, and the optimization compares topological layouts under a constant material amount. A full parametric optimization that simultaneously minimizes cost and maximizes performance is a promising direction.

## Figures and Tables

**Table 1:** Quantitative comparison of inspection results for the three sampled slabs.

Slab ID	Location	Max Crack Width (mm)	Crack Pattern	Concrete Strength (MPa)	Rebar Spacing (mm)
Slab A	2~3/M~N	0.32	Diagonal, fully developed	45.9	128
Slab B	7~8/X~Y	0.28	Diagonal, partially developed	46.5	125
Slab C	3~4/N~P	0.30	Diagonal, partially developed	44.8	130

Note: 2~3/M~N denotes the slab bounded by grid lines 2–3 and M–N; the same notation applies to 7~8/X~Y and 3~4/N~P.

**Table 2:** Roof slab thickness and mid-span deformation.

Member Name	Location	Design-Specified Roof Slab Thickness, mm	Measured Slab Thickness, mm						Top Slab Mid-Span Deformation, mm
			Test Point 1	Test Point 2	Test Point 3	Test Point 4	Test Point 5	Average Value	
Second-floor roof slab	2~3/M~N	150	159	157	150	148	156	154	2.23

Note: 2~3/M~N denotes the slab bounded by grid lines 2~3 and M~N.

**Table 3:** Concrete strength of roof slab.

Member Name	Area	Concrete Compressive Strength Conversion Value, MPa			Estimated Concrete Strength at Current Age, MPa	Design Strength
		Average Value	Standard Deviation	Minimum Value		
Second-floor roof slab	2~3/M~N	51.1	3.17	47.5	45.9	C30

Note: SD denotes the standard deviation of the rebound-hammer converted compressive strength; see Table 1 for the definition of 2~3 and M~N.

**Table 4:** Spacing of bottom rebar and concrete cover thickness of roof slab.

Member Name	Location	Rebar Orientation	Average Spacing of Bottom Rebar, mm		Concrete Cover Thickness, mm	
			Design	Measured	Measured Diameter of Each Rebar	Average Value
Second-floor roof slab	2~3/M~N	2↔3	120	128	21, 23, 23, 23, 22, 21, 20	22
		M↔N	100	108	13, 13, 15, 13, 14, 12, 17	14

Note: 2 ↔ 3 and M ↔ N denote the rebar direction between grid lines 2–3 and M–N, respectively; see Table 1 for the definition of 2~3 and M~N.

**Table 5:** Spacing of negative-moment rebars and concrete cover thickness of roof slab.

Member Name	Location	Average Spacing of Negative-Moment Rebars, mm		Concrete Cover Thickness of Negative-Moment Rebars, mm	
		Design	Measured	Measured Values of Each Rebar	Average Value
Second-floor roof slab	2~3/M~N	100/200	161	16, 18, 15, 19, 20, 16, 15	17
		100/200	160	20, 21, 19, 22, 18, 17, 21	20

Note: 100/200 means 100 mm in densified zones and 200 mm in non-densified zones; see Table 1 for the definition of 2~3 and M~N.

**Table 6:** Mid-span deflection results of slab under different conditions.

Mid-Span Deformation of Slab	Under Self-Weight, mm	Under Live Load of 4 kN/m^2^, mm	Code Limit, mm
Measured value	2.23	—	24.20
Simulated value	1.98	4.89	24.20

Note: The live-load deflection was not measured on site and is marked “—”; the deflection limit is 24.20 mm (L/250) [27].

**Table 7:** Comparison between the designed and actual measured concrete mix proportions.

Parameter	Designed Value	Actual Measured Value	Specification Limit	Status
Water-to-binder ratio (W/B)	0.45	0.55	≤0.55 (EN 206, XC3) [31]	At limit/Unfavorable
Cement content (kg/m^3^)	350	300	≥280 (EN 206, XC3) [31]	Qualified
Fly ash content (%)	15%	25%	≤25% (EN 450-1) [32]	At limit/Unfavorable
Fine aggregate mud content (%)	2.0%	4.5%	≤3.0% (ASTM C33) [33]	Exceeded

Note: Limits are taken from EN 206 [31], EN 450-1 [32], and ASTM C33 [33]; W/B denotes the water-to-binder ratio.

**Table 8:** Strengthening specifications of three CFRP schemes.

Layout Pattern	Longitudinal Layout Dimension, mm	Transverse Layout Dimension, mm	Total CFRP Consumption, mm^3^	CFRP Sheet Thickness, mm
Cross-shaped	2100	1500	526,050	0.167
Hash-shaped	1050 + 1050	900 + 600	526,050	0.167
Grid-shaped	150 × 14	150 × 10	526,050	0.167

Note: Grid dimensions are given as spacing × number of strips (e.g., 150 × 14 denotes 14 strips at 150 mm spacing); all three schemes have the same total CFRP volume (526,050 mm^3^).

**Table 9:** Scores of four-dimensional evaluation indicators.

Layout Pattern	Ultimate Bearing Capacity	CFRP Material Utilization Ratio	Crack Suppression Ratio	Relative Longitudinal Displacement
Cross-shaped	5.3	9.4	5.1	4.6
Hash-shaped	7.0	6.6	6.9	6.4
Grid-shaped	9.2	5.5	9.5	9.0

Note: Scores are normalized to a 1–10 scale using Equations (6) and (7); higher values indicate better performance. Relative longitudinal displacement is the normalized score of the negative indicator (interfacial slip), so a higher value denotes smaller slip.

## Data Availability

The original contributions presented in this study are included in the article. Further inquiries can be directed to the corresponding author.
